# Supplementary material for: Electrical and Thermal Conductivities of Single CuxO Nanowires
Source: Nanomaterials (Basel). 2023 Oct 25;13(21):2822. doi: 10.3390/nano13212822 (PMC10648451; doi:10.3390/nano13212822)
Supplement: Supplementary file 1 [file nanomaterials-13-02822-s001.zip › nanomaterials-2671300-supplementary.pdf]

Supplementary Materials

Electrical and Thermal Conductivities of Single Cu_xO Nanowires

Ivan De Carlo [1,2], Luisa Baudino [3], Petr Klapetek [4], Mara Serrapede [3], Fabio Michieletti [1,3], Natascia De Leo [1], Fabrizio Pirri [3,5,*], Luca Boarino [1], Andrea Lamberti [3,5] and Gianluca Milano [1,*]

[1] Advanced Materials Metrology and Life Sciences Division, Istituto Nazionale di Ricerca Metrologica (INRiM), 10135 Turin, Italy; ivan.decarlo@polito.it (I.D.C.); fabio.michieletti@polito.it (F.M.); n.deleo@inrim.it (N.D.L.); l.boarino@inrim.it (L.B.)
[2] Department of Electronics and Telecommunications, Politecnico di Torino, 10129 Turin, Italy
[3] Department of Applied Science and Technology, Politecnico di Torino, 10129 Turin, Italy; luisa.baudino@polito.it (L.B.); mara.serrapede@polito.it (M.S.); andrea.lamberti@polito.it (A.L.)
[4] Czech Metrology Institute, Okružní 31, 638 00 Brno, Czech Republic; pklapetek@cmi.cz
[5] Center for Sustainable Future Technologies @Polito, Istituto Italiano di Tecnologia (IIT), 10144 Turin, Italy
* Correspondence: fabrizio.pirri@polito.it (C.F.P.); g.milano@inrim.it (G.M.)

Table S1. Rietveld refinement main parameters for the cuprite and tenorite crystals.

**Cuprite**					**Tenorite**						
			**9007497**					**9016105**		**9014580**	
			**value**	**error**				**value**	**Error**	**value**	**error**
**Cell parameters**	**a**	Å	4.2865	$6 \cdot 10^{-4}$	**Cell parameters**	**a**	Å	4.6998	$1.3 \cdot 10^{-3}$	4.5978	$1.7 \cdot 10^{-3}$
						**b**	Å	3.4274	$9 \cdot 10^{-4}$	3.3233	$7 \cdot 10^{-4}$
	α	°	90			**c**	Å	5.1501	$1.4 \cdot 10^{-3}$	5.850	$1.2 \cdot 10^{-3}$
						**β**	°	99.389	$1.3 \cdot 10^{-2}$	99.500	$1.7 \cdot 10^{-2}$
**Crystalline size**		Å	754	38	**Crystalline size**		Å	461	18	2530	20
**Microstrain**			0.00310	$9 \cdot 10^{-5}$	**Microstrain**			0.00260	$1.4 \cdot 10^{-4}$	0.0035	$2 \cdot 10^{-4}$
**Density**		$g \cdot cm^{-3}$	6.0336		**Density**		$g \cdot cm^{-3}$	6.4553			
**Weight**		± 0.9 %	44.2 %		**Weight**		± 0.9 %	55.8 %			

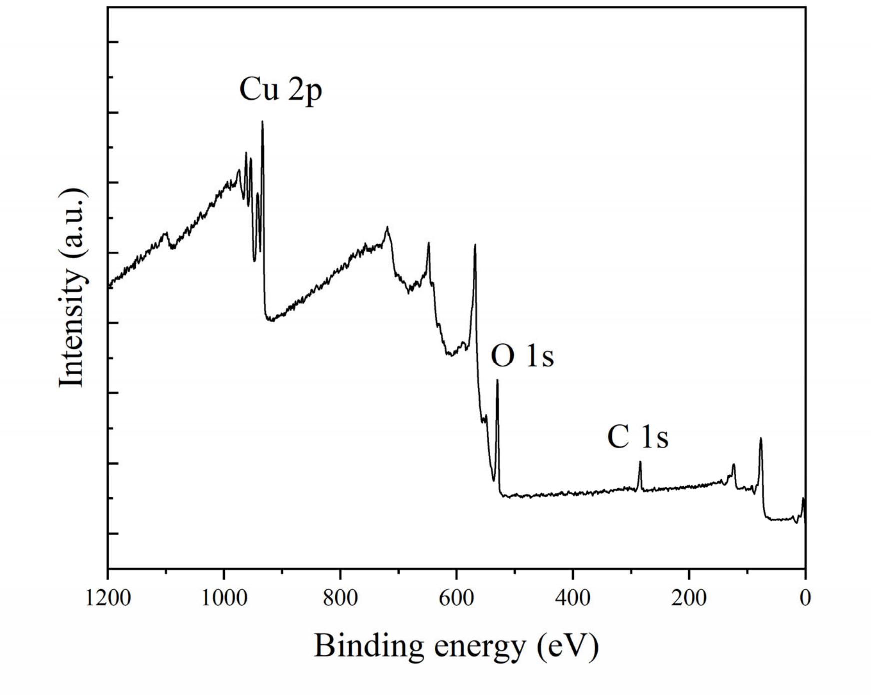

Figure S1. XPS survey scan of the NWs sample.

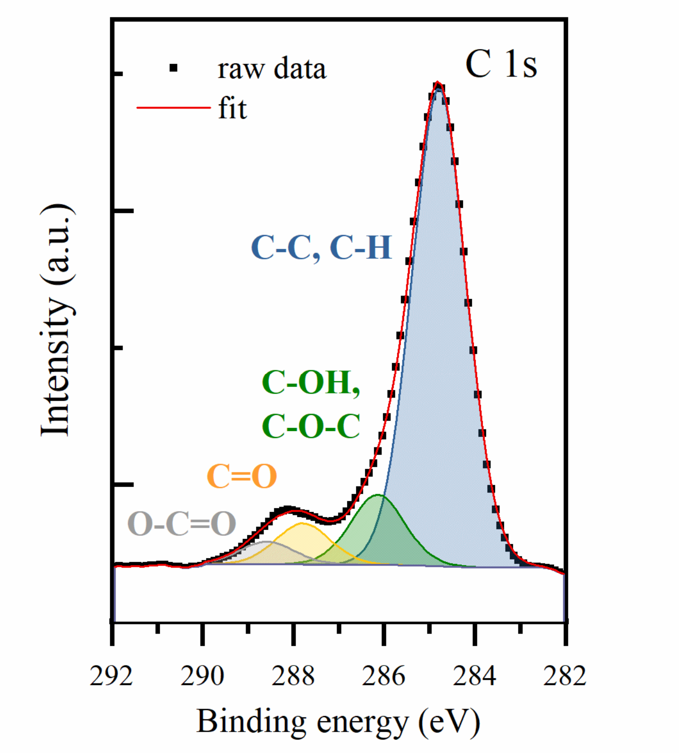

Figure S2. XPS HR scan of the C 1s region for calibration on the adventitious carbon, and deconvolution of the peak.
